# New Blocking Antibodies Impede Adhesion, Migration and Survival of Ovarian Cancer Cells, Highlighting MFGE8 as a Potential Therapeutic Target of Human Ovarian Carcinoma

**DOI:** 10.1371/journal.pone.0072708

**Published:** 2013-08-16

**Authors:** Lorenzo Tibaldi, Shirley Leyman, André Nicolas, Sofie Notebaert, Melissa Dewulf, Thu Hoa Ngo, Claudia Zuany-Amorim, Nathalie Amzallag, Isabelle Bernard-Pierrot, Xavier Sastre-Garau, Clotilde Théry

**Affiliations:** 1 Institut Curie, Centre de Recherche, Paris, France; 2 INSERM U932, Paris, France; 3 ThromboGenics, NV, Heverlee, Belgium; 4 Institut Curie, Hospital, Department of Tumor Biology, Paris, France; 5 CNRS UMR144, Paris, France; 6 CBT-507 IGR-Curie, Paris, France; Seoul National University, Republic of Korea

## Abstract

Milk Fat Globule – EGF – factor VIII (MFGE8), also called lactadherin, is a secreted protein, which binds extracellularly to phosphatidylserine and to αvβ3 and αvβ5 integrins. On human and mouse cells expressing these integrins, such as endothelial cells, phagocytes and some tumors, MFGE8/lactadherin has been shown to promote survival, epithelial to mesenchymal transition and phagocytosis. A protumoral function of MFGE8 has consequently been documented for a few types of human cancers, including melanoma, a subtype of breast cancers, and bladder carcinoma. Inhibiting the functions of MFGE8 could thus represent a new type of therapy for human cancers. Here, we show by immunohistochemistry on a collection of human ovarian cancers that MFGE8 is overexpressed in 45% of these tumors, and we confirm that it is specifically overexpressed in the triple-negative subtype of human breast cancers. We have established new *in vitro* assays to measure the effect of MFGE8 on survival, adhesion and migration of human ovarian and triple-negative breast cancer cell lines. Using these assays, we could identify new MFGE8-specific monoclonal antibodies, which efficiently blocked these three tumor-promoting effects of MFGE8. Our results suggest future use of MFGE8-blocking antibodies as new anti-cancer therapeutics in subgroups of ovarian carcinoma, and triple-negative breast carcinoma patients.

## Introduction

To develop as a full-blown tumor, a cell must not only acquire cell-autonomous properties of proliferation and resistance to programmed death, but also establish interactions with its microenvironment allowing its sustained proliferation, and avoiding its elimination [1]. Fibroblasts, endothelial cells forming blood vessels, and immune cells all exchange signals with the transformed cells through direct ligand-receptor interactions, as well as through soluble factors and extracellular membrane vesicles which act at a distance from the tumor cells. Tumors can thus secrete growth factors acting in an autocrine manner to sustain their survival, but also in a paracrine manner on the other cells of their microenvironment.

Milk Fat Globule – EGF – Factor VIII (MFGE8), also called lactadherin, is one of these secreted factors with pleiotropic potential functions. Originally cloned as a major protein of milk fat globules [2,3], the bovine, human (MFGE8) and mouse (Mfge8) proteins have been shown to contain two distinct functional domains: EGF-like domains including a RGD-containing sequence binding to αvβ3 and αvβ5 integrins, and Factor VIII-like (or discoidin) domains binding to phospholipids (phosphatidylserine and phosphatidylethanolamine). MFGE8/Lactadherin is thus bound non-covalently to lipids on extracellular vesicles, and interacts with target cells expressing αvβ3/5 integrins. MFGE8 binding to endothelial cells has been shown to promote VEGF-dependent survival and angiogenesis [4] as well as phagocytosis of apoptotic cells [5]. In the mouse, Mfge8 promotes phagocytosis of apoptotic cells by macrophages [6], and skews them to secrete tolerogenic cytokines [7]. On some tumor cells themselves, MFGE8 was shown to induce epithelial to mesenchymal transition [8,9], and/or to increase resistance to drug-induced apoptosis [10,11].

All these results highlight MFEG8 as a promising target for inhibitors that could be developed to limit tumor progression. In some types of human cancers, a pro-tumoral role of MFGE8 has been demonstrated, based on high overexpression during tumor progression, and/or on analysis of mouse models: these cancers include bladder carcinoma (our own work [12]), melanoma [8], and the triple-negative subtype of breast cancer [13]. However, in some other cancers, such as Hormone Receptor (HR) and/or HER2-expressing human breast cancers [13], MFGE8 is not overexpressed, and it seems instead to prevent tumor progression. Thus, generating new tools to inhibit the pleiotropic functions of MFGE8, as well as identifying the right human cancer targets of such tools, must be performed simultaneously if we hope to achieve efficient new therapies.

Here, by analysing MFGE8 expression in large arrays of human tumor biopsies, and by establishing new functional assays to measure the effects of MFGE8 and of new MFGE8-blocking antibodies on the physiology of tumor cells, we identified ovarian carcinoma, and confirmed triple-negative breast carcinoma as promising targets which could benefit from MFGE8-inhibiting therapies.

## Results

### MFGE8 overexpression in ovarian cancers

**Figure 1:**
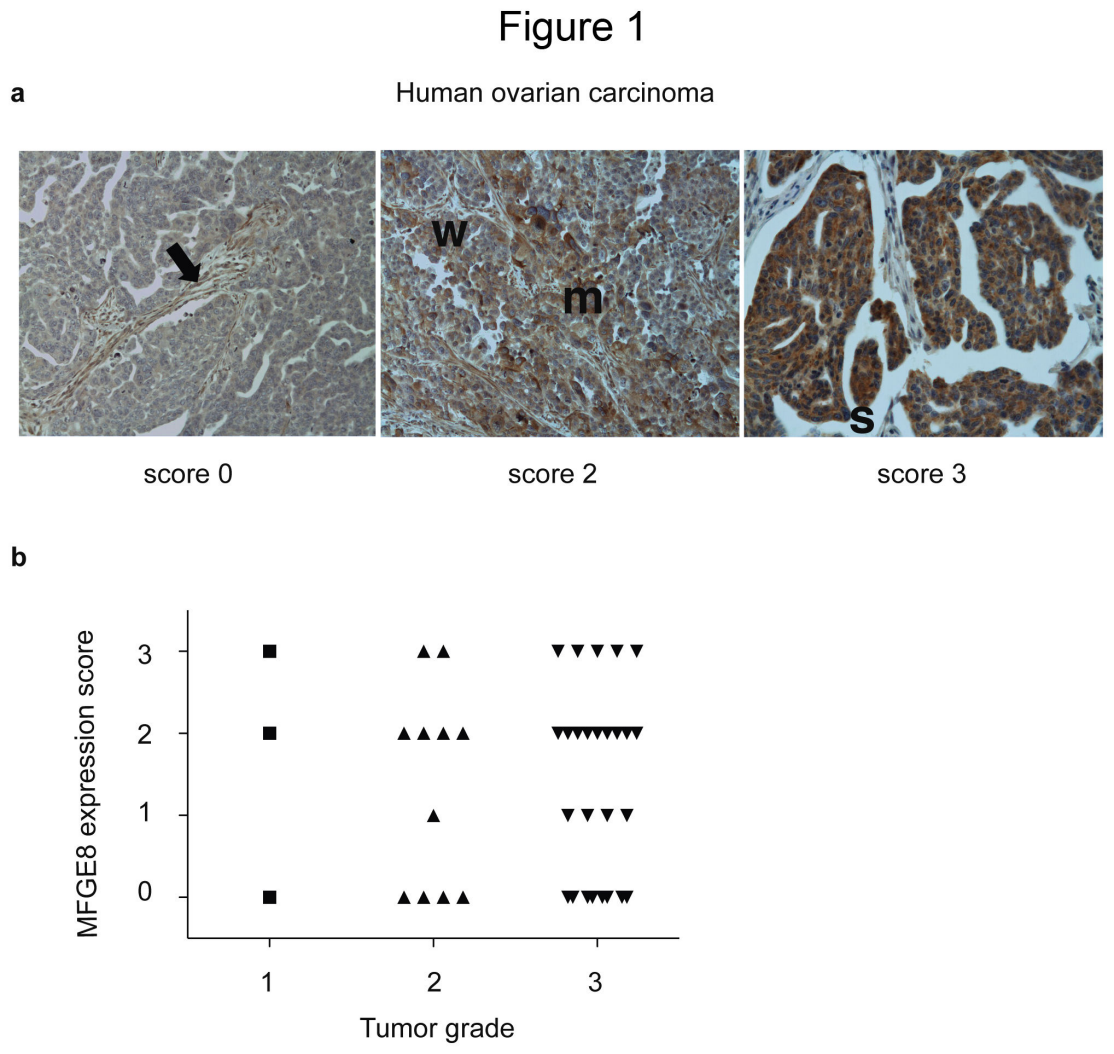
Expression of MFGE8 in biopsies of human ovarian carcinoma. (**a**) Examples of Human MFGE8 immunohistochemistry on ovarian carcinoma sections showing: no staining of tumor cells (score 0), but MFGE8-positive endothelial cells (black arrow) in the left panel. Areas of weak expression (w) and of medium expression (m) of MFGE8 in the same section, thus ranked as score 2 in the middle panel. Strong MFGE8 expression (s) throughout the tumor, ranked as score 3 (right panel). (**b**) Scoring of MFGE8 expression (y-axis) in 48 ovarian tumor biopsies from Institut Curie’s patients, as a function of tumor grade (x-axis). No statistically significant difference was observed in distribution of tumors overexpressing MFGE8 (score 2-3) or not (score 0-1) in grade 2 versus grade 3 tumors.

In a previous work, using publicly available mRNA expression data compiled in the oncomine website (*www.oncomine.org*), we observed a significant upregulation of the *MFGE8* gene at the transcriptomic level in a subset of human cancers, including ovarian serous adenocarcinomas [12]. Given the need for new treatments for this cancer, which often presents at advanced stage, we decided to further explore the roles of MFGE8 in ovarian carcinoma. To confirm the observation of *MFGE8* mRNA overexpression at the protein level, we used two tumor microarrays generated in-house, containing 50 biopsies from ovarian cancer patients of all grades and types (Table S1). Immunohistochemistry to detect MFGE8 in these tumors was performed using our previously described rabbit polyclonal anti-MFGE8 antibody [4], and analysis of the stainings was performed by a pathologist. As previously reported [4,12], MFGE8 was detected in blood vessels present within the tumors (arrow in Figure 1a). In addition, MFGE8-positive tumor cells themselves were observed in several biopsies. As shown in Figure 1a, the overall expression level of the protein was variable between individual tumors, ranging from absent (0), to very strong throughout the tumor (3). Ranking the tumors according to the level of MFGE8 protein (Figure 1b) showed that 45% (22/48) highly expressed MFGE8 (level 2-3). MFGE8-overexpressing tumors were of all grades (1 to 3, Figure 1b), of all types, and of various metastatic status (Table S1), thus MFGE8 overexpression could not be used as either a prognostic or a diagnostic marker of ovarian cancer. The high proportion of tumors overexpressing MFGE8, however, prompted us to further explore its value as a therapeutic target for ovarian cancers.

### Development of in vitro assays to quantify the effects of MFGE8 on ovarian cancer cells

**Figure 2:**
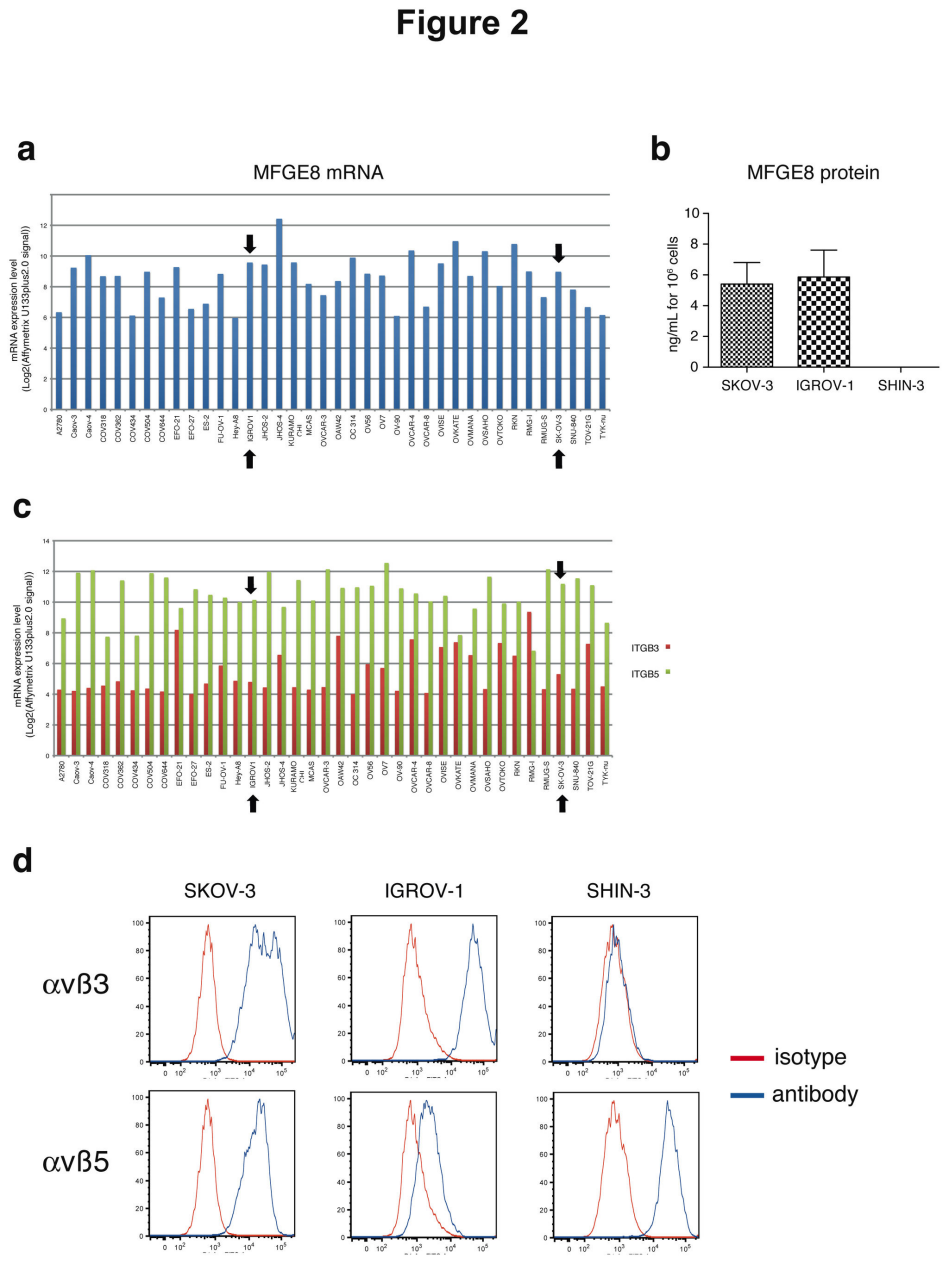
Expression of MFGE8 and its receptors in human ovarian cancer cell lines. (**a**) Analysis of mRNA expression levels (Log_2_(Affymetrix U133plus2.0 signal)) of *MFGE8* in public results of microarray data of the Broad-Novartis Cancer Cell Line Encyclopedia. The expression level threshold behind which it could be considered as noise (MAS5 “Absence” flag status, gene not expressed) is 6.1 for this probeset. Black arrows indicate the SKOV-3 and IGROV-1 cell lines. (**b**) Quantification of MFGE8 by ELISA in SKOV-3, IGROV-1 and SHIN-3 conditioned medium (24 hr). Secreted MFGE8 is expressed in ng/mL per 10^6^ cultured cells. Mean of two experiments + SD. (**c**) Analysis of mRNA expression levels (Log_2_(Affymetrix U133plus2.0 signal)) of *ITGB3* (red) and *ITGB5* (green) in public results of microarray data of the Broad-Novartis Cancer Cell Line Encyclopedia. The expression level threshold behind which it could be considered as noise (MAS5 “Absence” flag status, gene not expressed) is 4.8 for the *ITGB3* probeset. (**d**) FACS analysis of αvβ3 (upper panel) and αvβ5 (lower panel) integrins expression at the surface of SKOV-3, IGROV-1 and SHIN-3 cells. Specific antibody (blue line). Isotype control (red line).

Since MFGE8 has been shown in various cell types including some tumor cells, to promote adhesion, survival and/or epithelial-to-mesenchymal transition possibly leading to migration, we set up new *in vitro* assays to measure these physiological outcomes in the context of ovarian carcinoma. In most studies, these effects of MFGE8 were attributed to its binding on αvβ3 and/or αvβ5 integrins on target cells. In order to select cell lines to assess the effect of anti-MFGE8 therapies, we first analyzed the mRNA expression level of *MFGE8* and its receptors in a collection of human ovarian cancer cell lines. Public results from the Broad-Novartis Cancer Cell Line Encyclopedia were used for this purpose (http://www.broadinstitute.org/ccle/home). Out of the 38 tumor cell lines (Figure 2a), only 5 did not express *MFGE8* above the confidence level ((MAS5 “Absent” flag corresponding to an Affymetrix U133plus2.0 expression signal (Log_2_) lower than 6.1 for this probeset). We confirmed MFGE8 protein secretion by ELISA analysis of the conditioned medium of two of the mRNA-expressing cell lines: SKOV-3 and IGROV-1, and we identified another ovarian carcinoma cell line, SHIN-3 [14], which did not secrete detectable levels of MFGE8 *in vitro* (Figure 2b). The 38 tumor cell lines expressed the genes encoding αv (*ITGAV*, expression signal above 10 in most cases, data not shown), and β5 integrin chains (*ITGB5*, Figure 2c). By contrast, the Affymetrix signal for the β3 integrin chain (*ITGB3*) was only near the confidence level (expression signal = 4.8 for this probeset) for most cell lines (23/38 = 60,5%), including SKOV-3 and IGROV-1, suggesting weak or no expression of this integrin. By flow cytometry, however, both SKOV-3 and IGROV-1 displayed αvβ3 in addition to αvβ5 at their surface, whereas SHIN-1 only displayed αvβ5 (Figure 2d). We thus chose the SKOV-3 cell line as a representative example of a large proportion of the ovarian cancer cell lines (medium-to-high *MFGE8*, detectable *ITGB3* and *ITGB5*), and one susceptible to respond to MFGE8 since it expressed the known receptors for MFGE8, to develop the functional *in vitro* assays.

**Figure 3:**
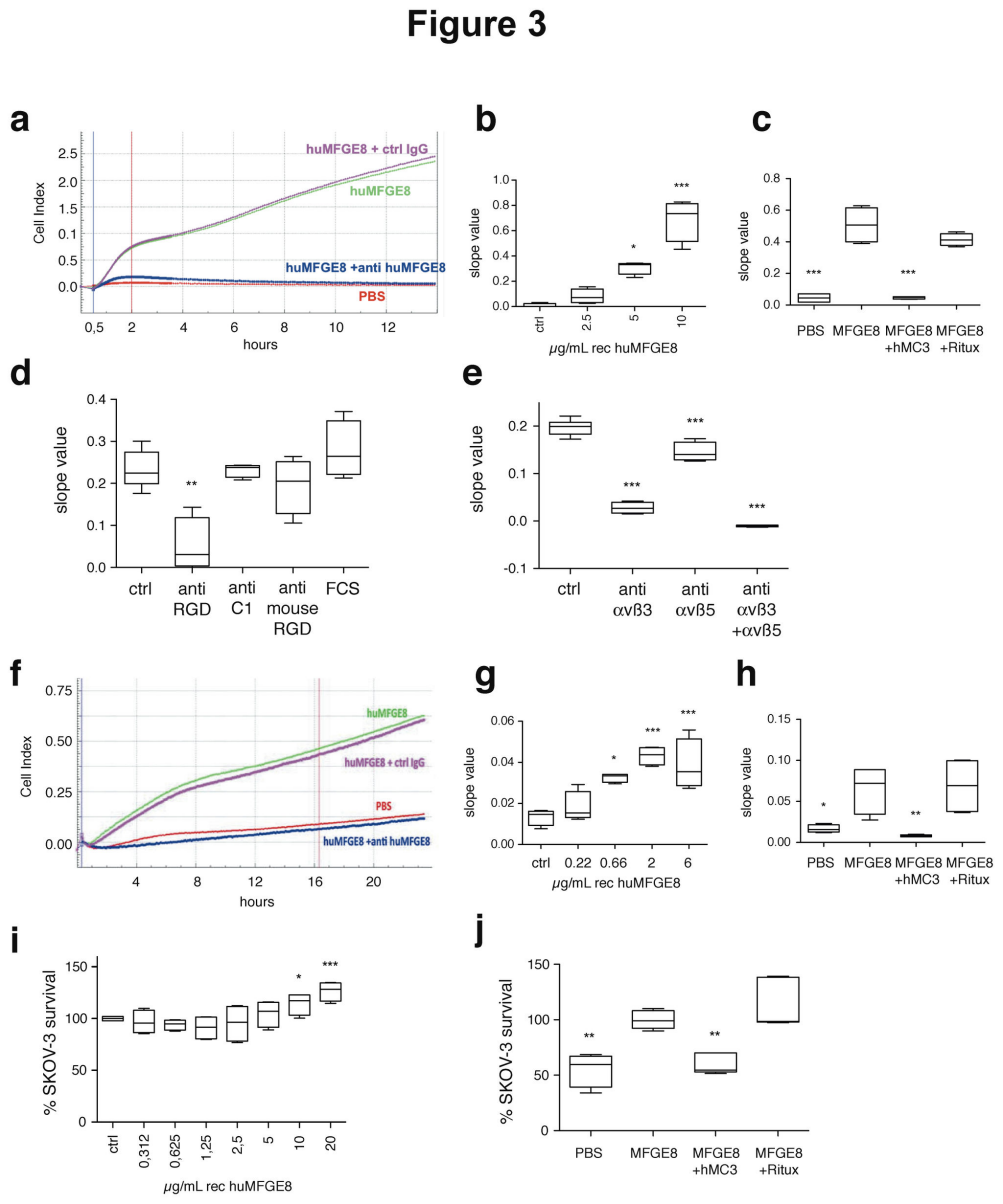
*In vitro* adhesion, migration and survival assays to measure effect of MFGE8 on SKOV-3 cells. (**a**–**e**) Assay for adhesion of SKOV-3 to MFGE8 using the xCELLigence system. (**a**) Example of cell index (CI) as a function of time, on PBS or human MFGE8 (huMFGE8), in the presence of anti-huMFGE8 or control IgG. Blue and red vertical lines indicate the time points used for calculating slope values. (**b**) Adhesion of SKOV-3 to MFGE8, as compared to PBS (Ctrl), quantified by slope value between 0 and 2h. (**c**) Inhibition of adhesion to 5 µg/mL MFGE8 by 10 µg/mL anti-huMFGE8 (hMc3) or a negative control (Rituximab, Ritux). (**d**) Effect on adhesion to 5 µg/mL MFGE8 (Ctrl) of polyclonal anti-huMFGE8 sera (1/100) specific for the RGD-domain (anti RGD), the C1 domain (anti C1) or the RGD domain of mouse Mfge8 (anti mouse RGD). (**e**) Inhibition of adhesion to 5 µg/mL MFGE8 (Ctrl) by anti-αvβ3 and/or -αvβ5 integrin antibodies (5µg/mL each). (**f**–**g**) Assay for migration of SKOV-3 using xCELLigence. Cells were seeded in the upper chamber, and MFGE8 and antibodies in the lower chamber of CIM-plates. (**f**) Example of cell index (y axis) as a function of time (x axis), in similar conditions as (**a**). Blue and red vertical lines indicate the time points used for calculating slope values. (**g**) Dose-dependent effect of MFGE8 on SKOV-3 migration, as compared to PBS (Ctrl), quantified by slope value between 0 and 18 hours. (**h**) Inhibition of migration to 5 µg/mL huMFGE8 by 10µg/mL anti-huMFGE8 hMc3, as compared to negative control (Ritux). (**i**–**j**) Survival assay of SKOV-3 cultured for 96 hours in the presence of 0.1% serum. Cell number is quantified as fluorescence arbitrary units (A.U.) as compared to the control condition corresponding to 100%. (**i**) Dose-dependent effect of MFGE8 on SKOV-3 survival. 100% = basal level of A.U. in the absence of exogenous MFGE8 (Ctrl). (**j**) Inhibition of survival in the presence of 10 µg/mL huMFGE8, by 10µg/mL hMc3 or the negative antibody. 100% = basal level of A.U. in the presence of MFGE8. Data are represented as Box-and-whiskers graphs showing the minimum (lower error bar), 25^th^ percentile-median-75^th^ percentile and maximum (upper error bar) values. N=4 (except 3j: N=5). *** = P<0.001; ** = P<0.01; * = P<0.05 compared to Ctrl = PBS in 3b, g; to 0.312 µg/ml MFGE8 in 3i; or to Ctrl = MFGE8 in 3c, d, e, h, j..

We developed 3 types of assays to investigate the effects of MFGE8 on adhesion, migration and survival of the tumor cells. The xCELLigence system (ACEA Biosciences), a device allowing real-time measurement of cell binding to a substrate, was used to quantify short-term adhesion to human recombinant MFGE8 (Figure 3a–e), and long-term transwell migration towards MFGE8 in the presence of fetal calf serum (FCS 0.1%) (Figure 3f–h). Classical fluorescence-based quantification of cell numbers was used to assess long-term survival in starvation conditions (Figure 3i, j).

The xCELLigence device continuously measures changes in impedance induced by cells when they encounter electrodes covering the bottom of a culture microwell (ePlate). The level of impedance signal is proportional to the number of cells and the intensity of their interaction with the substrate, and this signal can thus be converted to an arbitrary cell index value (Figure 3a, f). Efficiency of cell binding is calculated as the slope of impedance observed in the first two hours after cell seeding (Figure 3a–e). To measure adhesion of SKOV-3 to MFGE8, recombinant human MFGE8 was coated on the xCELLigence microwells, in conditions similar to those we had previously used to analyze binding of human endothelial cells to MFGE8 [4]. Figure 3b shows that MFGE8 allowed binding of the cells to the substrate in a dose-dependent manner. We tested the effects of previously described antibodies specific for human MFGE8 in this assay: rabbit sera generated in our laboratory, recognizing respectively either the RGD-containing integrin-binding domain [4] or the discoidin C1 domain of human MFGE8 (C. Théry, unpublished data), and a humanized version of the Mc3 monoclonal antibody described by Ceriani et al [15], (hMc3) [16] also recognizing the integrin-binding domain of MFGE8. Both the hMC3 antibody (Figure 3c) and the rabbit anti-RGD domain (Figure 3d) strongly inhibited cell adhesion, whereas neither a control humanized antibody (Ritux, Figure 3c), nor the rabbit anti-C1 domain serum, nor a rabbit serum to the RGD-domain of mouse Mfge8 (anti C1 or anti mouse RGD, Figure 3d) inhibited it. Finally, blocking antibodies to αvβ3 or αvβ5 inhibited binding, and their effects were additive (Figure 3e). Thus, adhesion of SKOV-3 to MFGE8 is mediated by interaction of the αvβ3/5-expressing cells with the RGD-containing domain of MFGE8.

The xCELLigence device was next used with electrode-coated Boyden chamber microwells (CIM Plates), which allows measurement of the impedance produced after migration of the cells to the lower side of an 8 µm pore-filter separating the upper from the lower chamber (Figure 3f). The ability of cells to migrate towards the MFGE8-containing lower compartment, in the presence of an identical small amount of FCS in both compartments (0.1%), was calculated as the slope of cell index between 0 and 18 hours after cell seeding in the upper chamber (Figure 3f–h). Figure 3g and 3h show that MFGE8 induced SKOV-3 migration in a dose-dependent manner, and that, as for the adhesion assay, hMC3 specifically inhibited this effect.

The third assay measured the effect of MFGE8 on survival of SKOV-3 cells cultured in starvation conditions (0.1% FCS). The presence of MFGE8 induced a dose-dependent increase of SKOV-3 survival, detectable four days after seeding (Figure 3i), which, although not very strong, was reproducible. This effect was also specifically blocked by the MFGE8-specific hMC3 antibody (Figure 3j).

### Generation of novel anti-MFGE8 monoclonal antibodies with anti-tumor properties

**Figure 4:**
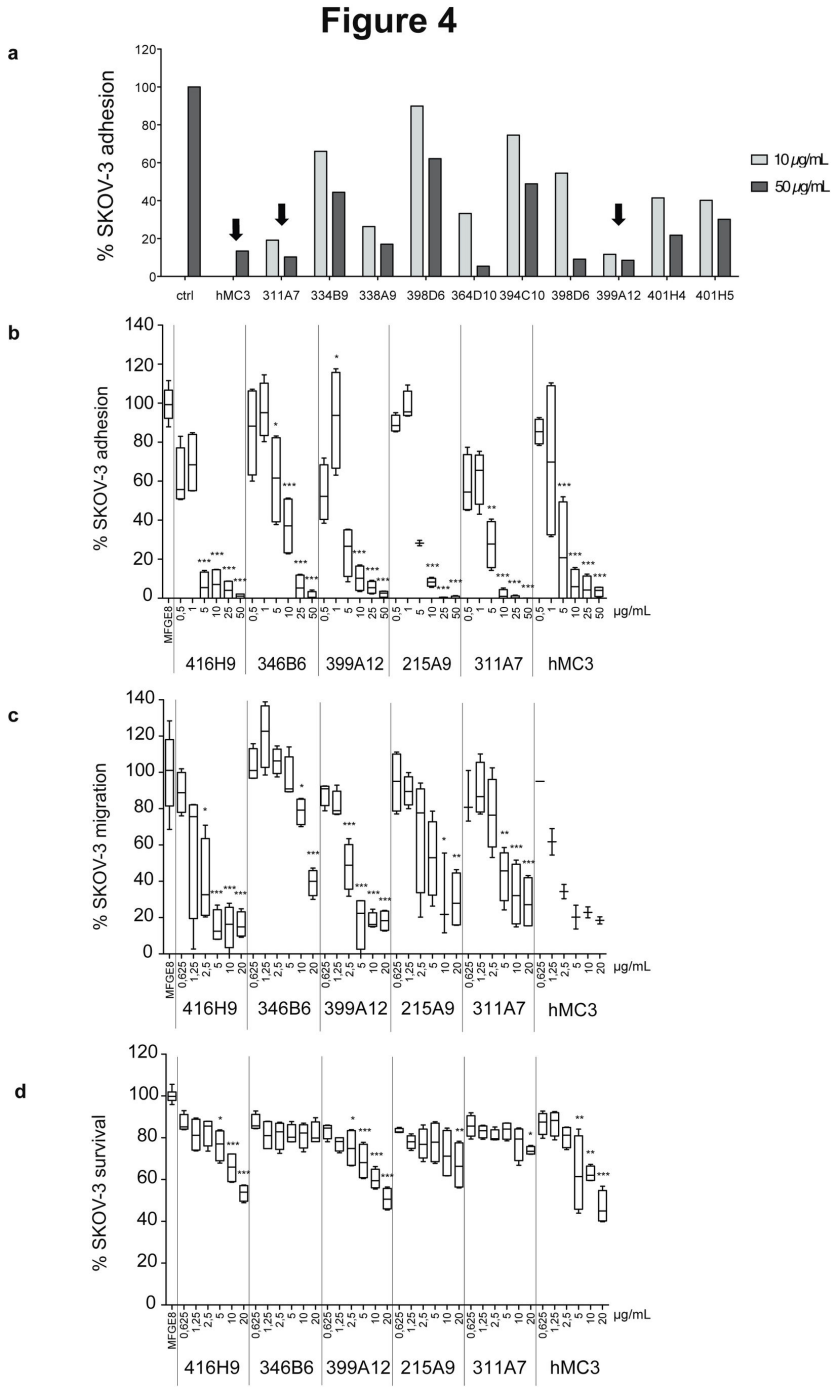
Identification of new antibodies blocking either adhesion, migration or survival induced by MFGE8. (**a**) Effect of 10 new anti-MFGE8 antibodies (10 or 50 µg/mL) on adhesion to MFGE8 (adhesion assay), as compared to hMC3. 100% adhesion corresponds to 5 µg/mL MFGE8 coating without antibodies (ctrl). Example of the screening experiments, performed once with each of the 40 new antibodies. Black arrows indicate the antibodies selected for further characterization. (**b**) Dose–response effect of the 5 selected antibodies, as compared to hMC3 in the adhesion assay. (**c**) Dose–response effect of the 5 selected antibodies, as compared to hMC3 in the migration assay. 100% migration corresponds to 5 µg/mL MFGE8 without antibodies. (**d**) Dose–response effect of the 5 selected antibodies, as compared to hMC3 in the survival assay. 100% survival corresponds to 10 µg/mL recombinant MFGE8 without antibodies. Box-and-whiskers graphs as in Figure 3. N=4 (except in (c) for 215A9 -10 µg/mL and 311A7 -0.625 µg/mL: N=3 and for hMc3: N=2; in (b) for 215A9 -5µg/mL: N=2). *** = P<0.001; ** = P<0.01; * = P<0.05 compared to the lowest antibody concentration (0.5 or 0.625 µg/ml) for each individual antibody in 4b, c, d.

To validate our new robust and reproducible *in vitro* assays as tools to identify new MFGE8 blocking agents, we used them to screen a large set of new anti-MFGE8 antibodies generated by immunizing our Mfge8-deficient mice [4,17] with recombinant MFGE8. In all assays, experimental conditions were compared to the cells grown in the presence of MFGE8, considered as 100% signal. The monoclonal antibodies displaying specific binding to human MFGE8 *in vitro*, and no binding to other proteins with common structural domains (i.e. vitronectin and Factor VIII) were tested in the SKOV-3 adhesion assay. The different antibodies displayed different inhibitory activities in this assay. Figure 4a shows examples of results of the initial screen (performed once) for 10 candidates. We selected five antibodies which displayed activities similar to the reference antibody hMc3 when used at 50 or 10 µg/mL.

To determine the efficacy of these 5 candidates, decreasing amounts of antibody from 50 to 0.5 µg/mL were used in the adhesion assay (Figure 4b). In the presence of 10 µg/mL of hMc3 or all clones, except for 346B6, less than 20% of cell adhesion was observed. In the case of 346B6 at 10 µg/mL, 40% of cell adhesion was detected (median of 4 measurements). In the presence of 0.5 µg/mL of three clones (416H9, 399A12 and 311A7), 60% cell adhesion was observed, whereas 90% adhesion was obtained with hMc3 at this concentration.

When tested in a dose–response setting in the migration assay (Figure 4c), all antibodies decreased migration when used at the highest concentration (20 µg/mL). Two clones (416H9, 399A12) significantly decreased migration when used at 2.5 µg/mL.

Finally, the antibodies were tested in the survival assay in the presence of 10 µg/mL MFGE8 (=100% survival in Figure 4d). 346B6, the least efficient clone in the other two assays, did not decrease survival at any concentration, two others induced at most 30% inhibition (=70% cell survival signal remained) when used at the highest concentration (2154A9 and 311A7), whereas the last two (416H9 and 399A12) were as efficient as hMc3, and decreased cell number to less than 50% of the MFGE8-alone treated cells (=50% survival).

The combined use of three complementary assays thus allows the clear selection of 2 antibodies, out of 40, as potentially the most efficient tools to simultaneously inhibit adhesion, migration and survival of MFGE8- and αvβ3/5-expressing ovarian carcinoma.

### Effect of MFGE8 and MFGE8-blocking antibodies on other ovarian carcinoma cells

**Figure 5:**
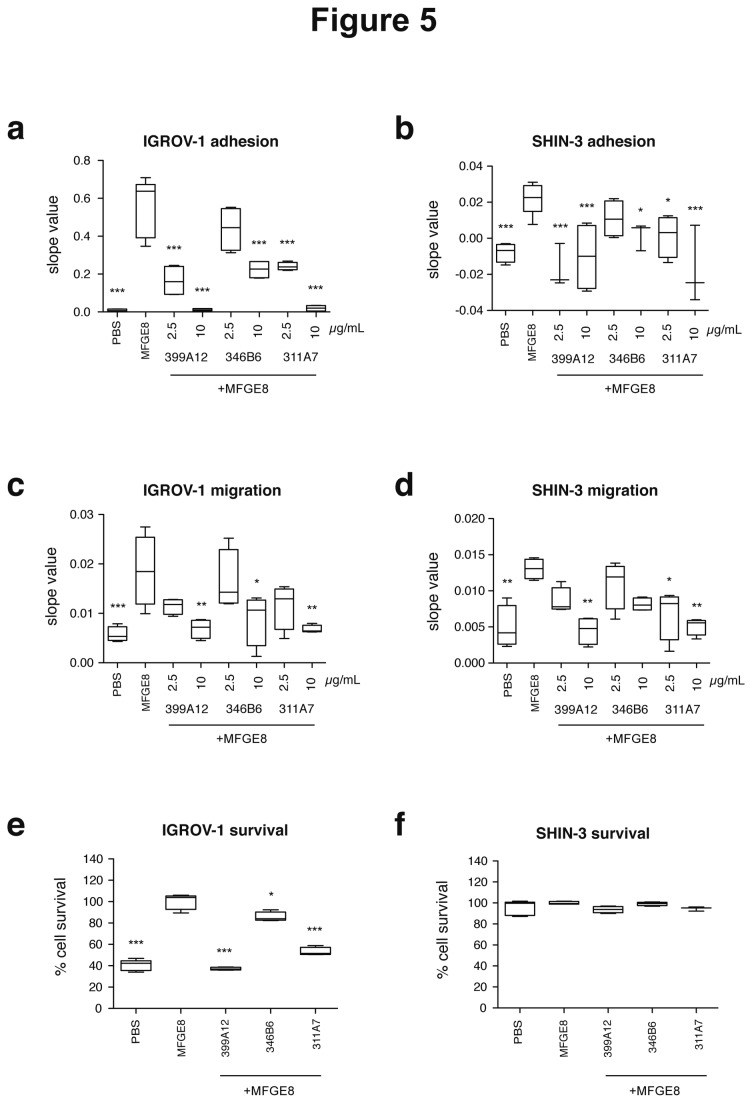
Effect of MFGE8 and anti-MFGE8 antibodies on IGROV-1 and SHIN-3 ovarian cancer cells. (**a**–**b**) Adhesion of IGROV-1 (a) and SHIN-3 (b) to 5 µg/mL MFGE8, as compared to PBS, quantified by slope value between 0 and 2h. The effect of three selected antibodies delivered at two doses (2,5 and 10 µg/mL) on MFGE8-coated wells is also shown. (**c**–**d**) IGROV-1 (c) and SHIN-3 (d) migration assay to 5µg/mL MFGE8 as in Figure 3-4, and inhibition by antibodies as in panels a and b for adhesion. Slopes values calculated between 0 and 18 hours. (**d**–**e**) IGROV-1 (d) and SHIN-3 (e) survival assay in the presence of 10µg/mL MFGE8, as in Figure 3-4, and inhibition by 2,5 or 10µg/mL antibodies. 100% survival corresponds to 10 µg/mL MFGE8 without antibodies. Box-and-whiskers graphs as in Figure 3. N=4 (except in (b): 399A12 -2.5 µg/mL, 346B6 -10 µg/mL, 311A7 -10 µg/mL: N = 3; in (e, f) PBS and MFGE8 : N=5; in (f) MFGE8 + 311A7: N=3). *** = P<0.001; ** = P<0.01; * = P<0.05, compared to Ctrl = MFGE8.

To determine whether the results obtained with SKOV-3 were representative of other ovarian carcinoma cell lines, we performed the same three assays using IGROV-1 and SHIN-3, which, as shown in Figure 2, either expressed MFGE8 and αvβ3/5 integrins at levels similar to SKOV-3 (IGROV-1), or did not express MFGE8 or the αvβ3 integrin (SHIN-3). Three antibodies representative of the three types of efficiencies observed in SKOV-3 were used here: 399A12, 311A7 and 346B6, respectively high, medium, and low blocking efficiencies. In the xCELLigence-based adhesion assay (Figure 5a, b), SHIN-3 gave a 20-times lower impedance signal than SKOV-3 or IGROV-1 (compare slope values in Figure 5b and 5a), but as in SKOV-3, adhesion was increased for both cell lines in the presence of 5 µg/mL MFGE8, and inhibited in a dose-dependent manner by the 3 antibodies. In the migration assay (Figure 5c, d), where SHIN-3 displayed a rate of migration similar to SKOV-3 and IGROV-1, the three antibodies also inhibited MFGE8-dependent migration of IGROV-1 and SHIN-3 in a dose-dependent manner, and with the same high-, medium- and low efficiency as in SKOV-3. By contrast, the survival assay (Figure 5e, f) showed a different effect of MFGE8 on the three cell lines: SHIN-3 did not display any increased survival in the presence of MFGE8, nor any effect of the MFGE8-blocking antibodies in this assay, whereas IGROV-1 behaved like SKOV-3.

### MFGE8 overexpression in triple negative breast cancers

**Figure 6:**
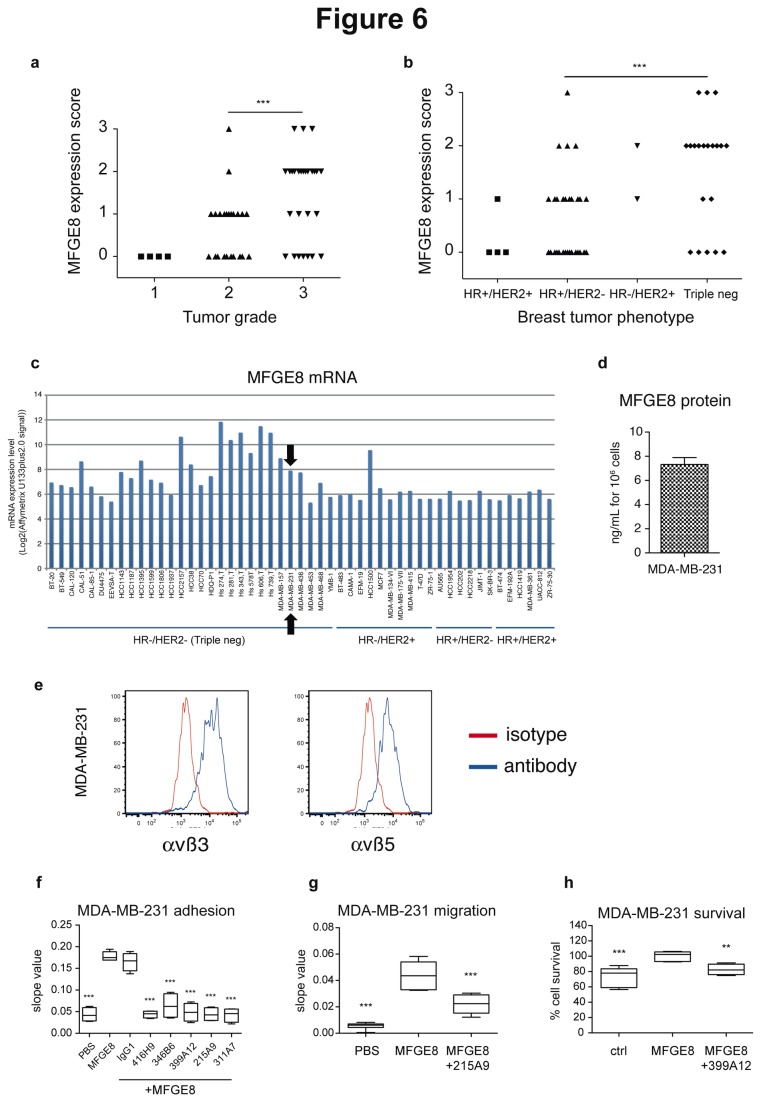
Expression of MFGE8 in human breast carcinoma and effect of MFGE8 on MDA-MB-231 cells. (**a**–**b**) Scoring of MFGE8 expression, analyzed by immunohistochemistry as in Figure 1, in 59 breast tumor biopsies from Institut Curie’s patients, as a function of tumor grade (a) or phenotype in terms of Hormon Receptor (HR) or HER2 expression (**b**). ***: P = 0.0001 for distribution of tumors overexpressing (score 2-3) or not (score 0-1) MFGE8 between grade 2 versus grade 3 tumors (a), or between HR+/HER2- versus triple-negative tumors (b). (**c**) Analysis of mRNA expression levels (Log_2_(Affymetrix U133plus2.0 signal)) of *MFGE8* in public results of Affymetrix microarray data of the Broad-Novartis Cancer Cell Line Encyclopedia as in Figure 2. Black arrow indicates the MDA-MB-231 cell line (HR-/HER2- triple negative phenotype) selected for subsequent experiments. (**d**) Quantification of MFGE8 by ELISA in MDA-MB-231 conditioned medium (24 hr). Secreted MFGE8 is expressed in ng/mL per 10^6^ cultured cells. Mean of two experiments + SD. (**e**) FACS analysis of αvβ3 (left panel) and αvβ5 (right panel) integrin expression at the surface of MDA-MB-231 cells. Specific antibody (blue line). Isotype control (red line). (**f**) MDA-MB-231 adhesion assay on 5µg/mL MFGE8, as in Figure 3-5, and inhibition by 10µg/mL hMC3, represented as slope values between 0 and 1 hour. (**g**) MDA-MB-231 migration assay to 5µg/mL MFGE8 as in Figure 3-5, and inhibition by 10µg/mL 215A9 antibody. Data represented as slope values calculated between 0 and 18 hours. (**h**) MDA-MB-231 survival assay in the presence of 10 µg/mL MFGE8, as in Figure 3-5, and inhibition by 10 µg/mL 399A12. 100% survival corresponds to 5µg/mL MFGE8 without antibodies. Box-and-whiskers graphs as in Figure 3. N=4 (except in (g): PBS N=6, MFGE8 + 215A9 N=5, and in (h): ctrl N=6, MFGE8: N=5). ***: P<0.001, **: P<0.01, compared to Ctrl = MFGE8.

Finally, to determine whether new MFGE8-blocking agents, such as the antibodies identified above, could be used for treatment of other tumors, we analyzed MFGE8 expression by immunohistochemistry in our collection of breast tumor biopsies generated from patients treated at Institut Curie (Table S2). MFGE8 was more strongly expressed in patients with advanced grades of breast cancer (Figure 6a), and more specifically in the triple-negative HR-/HER2- subtype of these cancers (which are in majority of grade 3): 13/20 = 65% of triple-negative biopsies displayed MFGE8 levels ranked as 2 or 3, whereas only 5/36 = 13.8% of the hormone receptor (HR) and/or HER2 positive tumors displayed levels 2 or 3 of MFGE8 expression (Figure 6b). These observations thus confirm the results recently published by Yang and collegues [13].

Consistently, analysis of the Broad-Novartis Cancer Cell Line Encyclopedia expression data of breast cancer cell lines showed expression of *MFGE8* above the confidence level (Log_2_ Affymetrix U133plus2.0 signal of MAS5 “absence” flag = 6,1 for *MFGE8*) in the majority of triple-negative breast cells (24/29 = 82%), whereas only 2/23 = 9% of the HR and/or HER2+ cells expressed the gene (Figure 6c). Finally, we tested the effects of MFGE8 and the blocking antibodies on adhesion, migration and survival of the triple-negative MDA-MB-231 cell line, which secretes MFGE8 and expresses αvβ3 and αvβ5 integrins like SKOV-3 and IGROV-1 (Figure 6d, e). As shown in Figure 6f–h, MDA-MB-231 displayed MFGE8-induced adhesion (Figure 6f), migration (Figure 6g), and survival (Figure 6h). Furthermore, the anti-MFGE8 antibodies displayed similar effects on MDA-MB-231 as they did on SKOV-3 cells.

Our results thus confirm MFGE8 as a promising target for an antibody-based strategy aiming at blocking tumor growth of triple-negative breast cancers.

## Discussion

The results presented here highlight a new type of cancer as a potential target for MFGE8-blocking therapies: ovarian carcinoma. Furthermore, we describe new assays adaptable to large-scale screening, and to cancer cell lines of different tissue origins, which will allow identifying the most useful MFGE8-blocking agents to be next used in pre-clinical *in vivo* tumor growth assays.

The combined analysis of 3 physiological effects of MFGE8/lactadherin on tumor cells, i.e. adhesion, migration and survival, allowed us to rank 5 different MFGE8-specific antibodies, and to compare them to the reference antibody hMc3. Mc3 was described 30 years ago for its ability to bind to circulating mammary epithelial cell antigens in cancer patients [18]. It was soon after shown to inhibit growth of human breast tumors in Nude mice either alone [15] or, more efficiently, as a conjugate with ^131^I [19]. The antigen recognized by Mc3 was cloned as MFGE8 (at the time called BA46) a few years later [3], and epitope mapping identified the RGD-containing EGF domain as target of Mc3, whereas another antibody, Mc8, recognized an epitope in the C2 domain and did not display antitumor activity *in vivo* [20]. In our three assays, Mc3 efficiently inhibited the effects of recombinant MFGE8, suggesting that the RGD domain and its interaction with αvβ3/5 integrins are required for adhesion, migration through transwell, and cell survival. Since we observed that, among our two anti-MFGE8 rabbit antisera, only the one recognizing the RGD domain blocks adhesion, we can exclude a role of other domains of MFGE8 in this type of function. But we cannot exclude that migration and survival could also be mediated by other types of interactions of MFGE8 with the target cell, possibly via another receptor, as suggested for sperm binding to egg mediated by SED1 (another name of MFGE8) [21]. Among the 40 new MFGE8-specific monoclonal antibodies generated during this project, some may display an RGD-independent migration or survival inhibiting activity, but we did not analyze them, since the first round of screening and selection was based exclusively on the adhesion assay.

The strength of our assays is the combined analysis of different functional effects of MFGE8. Obviously, the effect of MFGE8 on cell survival was weaker than on migration or adhesion, but the survival assay was the most discriminant. It allowed us to identify with certainty two of the new antibodies displaying similar efficiencies as hMc3 (416H9 and 399A12), whereas two other antibodies (311A7 and 215A9), although they very efficiently decreased cell adhesion and migration, only poorly inhibited survival. Interestingly, sequencing the variable domains of these five antibodies (PCT/EP2013/056057) revealed identical sequences of both heavy and light chains of clones 416H9 and 399A12, which behaved identically in all assays, thus further confirming the validity of the combined *in vitro* assays. This result suggests that, if used *in vivo*, 311A7 and 215A9 would induce less pleiotropic anti-tumor effects. Measuring the effects of these antibodies on SKOV-3 cells implanted in immunodeficient mice must now be done.

The survival assay also provides another interesting information: as opposed to both the adhesion and the migration assays, it worked only on 3 out of the 4 tumor cell lines used here. SHIN-3 is the only one of the four cell lines which does not express αvβ3, and we hypothesize that αvβ3 could be required for the survival function of MFGE8. However, other surface receptors or secreted factors could also interfere with MFGE8 binding, and may differ between SHIN-3 and the other cell lines. Exploring these other factors, and the respective roles of the two integrins on MFGE8-dependent physiological effects, will be further analyzed, and may provide future important criteria for inclusion of patients in MFGE8-blocking therapies.

We want to stress that the survival effect of MFGE8, although weak, is highly relevant to the *in vivo* situation. It was observed only after several days in culture (at least 3), and it was not visible if FCS concentrations of 1% or 10% were included in the culture medium (data not shown). Thus MFGE8 promotes survival only in the presence of very low FCS levels, mimicking the starvation conditions inside a poorly vascularized tumor. By contrast, the migration-promoting activity of MFGE8 would be more relevant at the tumor edges, where tumor cells interact with the extracellular matrix. MFGE8 has also been reported to protect melanoma cell lines from stressful conditions induced by chemotherapy leading to apoptosis [8]. Thus, blocking MFGE8 may be also a promising approach in combination with some chemotherapies to allow destruction of the most resistant tumor cells.

Our results also show a new unexpected physiological effect of MFGE8 on the ovarian carcinoma cell line: chemotaxis. MFGE8 induced cell migration in the transwell CIM-plate assay, only when added at a distance from the cells, in the lower chamber compartment. We did not observe any migration if MFGE8 was included with the cells in the upper chamber (data not shown). Thus rather than inducing migration by inducing acquisition of a mesenchymal state [9], or by binding to phosphatidylserine at the cell surface, as described for intestinal epithelial cells [22], MFGE8 here is instead a chemoattractant for tumor cells. How this function is regulated, and whether the receptors involved are also αvβ3/5 integrins will have to be evaluated, but it seems similar to a recently described effect of another ligand of αvβ3/5 integrins, osteopontin, on lung cancer [23].

Finally, we focused here on analyzing the effects of MFGE8 on the tumor cells themselves, but within the tumor microenvironment, both endothelial cells and immune cells such as macrophages, dendritic cells and possibly others, express αvβ3/5 integrins, and may be affected by MFGE8. We have previously shown that human MFGE8 promotes AKT signaling in human endothelial cells [4], both directly, and by potentiating VEGF-induced signaling : MFGE8 thus represents another pro-angiogenic molecule in the tumor microenvironment. The effects of MFGE8 on immune cells, on the other hand, have been clearly demonstrated in mouse models, but, to our knowledge, have not been demonstrated in human. In mice, Mfge8 promotes phagocytosis of apoptotic cells by endothelial cells [5] or macrophages [6] and concomitantly induces them to secrete tolerogenic cytokines [7], which should help tumors to escape attack by the immune system. In addition, mouse macrophages within transplanted tumors or after activation by inflammatory signals secrete high levels of Mfge8 [6,10], which have been shown to help tumor cells, especially cancer stem cells, resist apoptosis [10]. But whether human MFGE8 also induces tolerogenic macrophages in the context of tumors, is not known. The human MFGE8 displays several structural differences with its mouse counterpart, such as containing only one instead of two EGF domains, and no splice variant containing a Proline/Threonine rich domain between the EGF and discoidin domains. Whether human MFGE8 displays tolerogenic potential as described for mouse Mfge8 is not known. Thus determining the role of MFGE8 and of the MFGE8-blocking agents in the physiology of non-tumoral cells present within the human tumor microenvironment will be another important issue to address, to use them in the best conditions in clinical settings.

## Materials and Methods

### Immunohistochemistry on human tumor biopsies

Paraffin-embedded breast and ovarian tumor tissues of patients treated at Curie Hospital in the period between 1993 and 2003 were used to generate tumor microarrays slides containing two cores of 1mm diameter of 25 different biopsies per slide. Immunohistochemistry to label MFGE8 was performed after antigen unmasking by heat-induced epitope retrieval and blocking with horse serum, by incubating slides with peptide-purified anti-human MFGE8 rabbit antibody at 9 µg/mL. After washing and incubation with HRP-coupled secondary antibody, slides were revealed with Vectastain *Elite* ABC kit (Vector) before counterstaining with hematoxylin.

Scoring of the level of MFGE8 expression was performed by a trained pathologist. Levels were defined as : no expression = 0 ; faint expression = 1 ; medium-level expression throughout the tumor or high-level expression in defined area of the tumor = 2 ; overall strong expression = 3.

### Ethics Statements

Tumors were obtained from the Biological Resource Center (BRC) of Institut Curie. The tumor collections are registered by the French Ministry of Health under n° DC-2008-57, with authorization for distribution n° AC-2008-59. According to French regulations, patients were informed of research performed using the biological specimens obtained during their treatment and did not express opposition.

### Cell culture and reagents

The cancer cell lines (SKOV-3, IGROV-1, SHIN-3, MDA-MB-231) used in this work were cultured in RPMI or DMEM Glutamax (for MDA-MB-231) medium (Gibco) supplemented with Penicillin, Streptomycin and 10% Fetal Calf Serum (FCS, Lonza). SKOV-3 were purchased from ATCC, IGROV-1, SHIN-3 and MDA-MB-231 were provided by Dr V. Soumelis (Institut Curie). Recombinant MFGE8 was purchased from R&D systems. Anti-human αvβ3 (MAB1976Z) and αvβ5 (MAB1961Z) antibodies (Millipore) were used as blocking antibodies, and to analyze cell surface expression by Flow Cytometry, using a MacsQuant (Miltenyi Biotech) flow cytometer, and FlowJo software. Sandwich ELISA to detect human MFGE8 was purchased from R&D, and used to quantify MFGE8 in conditioned medium from 5x10^5^ tumor cells seeded in 34 mm plates for 24h. MFGE8 concentration was normalized to the number of secreting cells.

### Anti-MFGE8 antibodies

The humanized hMC3 antibody (generated as described by Couto and colleagues [16]), and the new murine monoclonal anti-human MFGE8 antibodies obtained by immunizing Mfge8-deficient mice [4,17] with recombinant human MFGE8 and murine Mfge8 protein (R&D Systems) were produced at ThromboGenics NV. Clones of monoclonal antibody-producing cells were screened by ELISA for specific binding to human MFGE8, and no binding to human vitronectin. Control antibodies for the anti-MFGE8 monoclonal antibodies were respectively, humanized anti-CD20 (Rituximab), obtained from the pharmacy of Institut Curie (MabThera, Roche), and various mouse IgG non-specific for MFGE8, provided by ThromboGenics NV. Three rabbit sera specific for human MFGE8 or mouse Mfge8 were generated in 1998 by synt:em, Inc, for our laboratory, by immunizing rabbits with either a peptide covering the RGD sequence of human MFGE8 (CEEISQEVRGDVFPSY), of mouse Mfge8 (CLVTLDTQRGDIFTEY) (described in [4,17]), or two peptides from the C1 domain of mouse and human MFGE8 (CEYLKTFKVAYSLDG and CVTGIITQGARDFG). The anti-human sera recognized recombinant MFGE8 and a single band in human milk by Western Blot (not shown).

### Adhesion assay

The adhesion assay was developed using the xCELLigence system (ACEA Biosciences), allowing real-time monitoring of cell interaction with electrodes integrated in flat-bottomed tissue culture microplates (E-Plate, ACEA Biosciences). The resulting impedance depends on the number of adherent cells and on the quality of their interaction with the electrodes, and allows the calculation of an arbitrary Cell Index (CI) value (RTCA software, ACEA Biosciences). The slope of CI variation over time (Figure 3a, f), calculated by the RTCA software, quantifies the efficiency of cell binding to the wells.

E-Plate wells were coated overnight at 4°C with 5 µg/mL recombinant human MFGE8 diluted in PBS (or different doses in Figure 3b). After two washes with PBS, wells were saturated for 1 hour with 1% BSA in PBS. After one last wash in PBS, 50 µL serum-free RPMI or DMEM was added per well and E-Plates were set in the xCELLigence device in a 37°C and 5% CO_2_ incubator for 1 hour to equilibrate wells and electrodes. Background impedance was measured after this step (= t0). E-Plates were taken out of the xCELLigence device, 50 µL/well of antibodies (diluted at 3x final concentration in RPMI) were added, followed by 2x10^4^ cells in 50 µL serum-free medium/well. E-Plates were transferred back onto the xCELLigence instrument, which was left again to equilibrate for 20 min before the beginning of impedance recording. Impedance was then measured every 15 seconds for the first 4 h, followed by every 5 minutes for the next 44 hours. Data were plotted using the RTCA software as CI value over time, and the slope of CI between the first time of recording after cell seeding and the time required for impedance to reach a first plateau (90 minutes for ovarian cancer cell lines or 60 min for MDA-MB-231) was calculated.

### Migration assay

For the migration assay, the xCELLigence system was used with CIM tissue culture microplates (ACEA Biosciences), i.e. Boyden chamber-type tissue culture wells, with a lower and an upper compartment separated by a 8 µm pore-containing membrane, with the impedance-measuring electrodes at its lower side. The bottom chamber of CIM-plate wells were loaded with 160 µL medium-0.1% FCS containing MFGE8 and/or antibodies at the final concentration, the transwell insert was positioned and 50µL/well of medium-0.1% FCS was seeded in the upper chamber. CIM-Plates were transferred onto the xCELLigence instrument and left to equilibrate for 1 hr, at which time background impedance was measured (= t0). Plates were taken out and 50 µL of medium-0.1% FCS containing 2x10^4^ cells were loaded in each well. Plates were placed back in the xCELLigence device, which was set to measure impedance every 5 minutes for the next 72 hours. Data were analyzed as above, and the slope of CI between 0 and 18 h was chosen as the shortest and most discriminant time point to evidence the differences in the different conditions.

### Survival assay

To measure survival in low-serum conditions, 2x10^4^ cells were seeded in tissue culture 96-well plates, in 50 µL medium-0.1% FCS/well. MFGE8 and antibody dilutions were added as 2x concentrated solutions in RPMI-0.1% in 50 µL/well each. Cells were kept in culture at 37°C and 5% CO2 for the next 96 h. Cell viability was determined by adding 10 µL of Cell titer blue reagent (Promega) and incubating plates for another 2 h at 37°C, before reading fluorescence at 616 nm (emission) after excitation at 544 nm.

### Statistical analyses

Statistical analysis on the distribution of human tumor biopsies between those overexpressing MFGE8 (level 2 or 3) or not (level 0 or 1) was performed using Fisher’s exact test (Figures 1b, 6a-b). Statistical analyses on measurements obtained in 2 independent experiments of the migration, adhesion, and survival assays were performed using 2-way ANOVA followed by Tukey’s post-hoc test, with p-values adjusted for multiple testing (Figures 3, 4, 5, 6f–h). The significance level was set at 5%. Analyses were performed using the R software (version 2.13.1, http://www.R-project.org/) [24].

## Supporting Information

Table S1 **List of Ovarian carcinoma biopsies used to generate the tumor microarray used for histology.**
(DOCX)

Table S2 **List of breast carcinoma biopsies used to generate the tumor microarray used for histology.**
(DOCX)
