# Supplementary material for: New Blocking Antibodies Impede Adhesion, Migration and Survival of Ovarian Cancer Cells, Highlighting MFGE8 as a Potential Therapeutic Target of Human Ovarian Carcinoma
Source: PLoS One. 2013 Aug 16;8(8):e72708. doi: 10.1371/journal.pone.0072708 (PMC3745384; doi:10.1371/journal.pone.0072708)
Supplement: Table S1 — List of Ovarian carcinoma biopsies used to generate the tumor microarray used for histology. (DOCX) [file pone.0072708.s001.docx]

**Table S1 : List of Ovarian carcinoma biopsies used to generate the tumor microarray used for histology**

abbreviations**:** Treatment: 5FU= Fluouracile, MTX= Methotrexate; Response: CR= Complete Response; PR= Partial Response; SR= Stable Response; TP= Tumor Progression. FIGO stage: Stage I= tumor confined to the ovaries; Ia= one ovary affected, ovary capsule intact, no tumor on ovary’s surface; Ib= both ovaries affected, capsule intact, no tumor on ovary’s surface; Ic= tumor limited to one or both ovaries and ovary present at least one of the following: capsule ruptured, tumor on ovary’s surface, malignant cells in ascites. Stage II= tumor growth in one or both ovaries, with pelvic extension. IIa= Extension and/or metastasis to the uterus and/or tubes; IIb= extension to other pelvic tissues; IIc= as in IIa or IIb but possibly showing also capsule ruptured and/or tumor on ovary’s surface and/or malignant cells in ascites; Stage III= regional metastasis; IIIa= microscopic peritoneal metastasis; IIIb= macroscopic peritoneal metastasis <2cm; IIIc= macroscopic peritoneal metastasis >2cm and/or positive retroperitoneal or inguinal nodes; Stage IV= distant metastasis.

| **TMA sample** | **Carcinoma type** | **Grade** | **MFGE8 score** | **FIGO stage** | **Metastatic site** | **Treatment** | **Reponse** |
| --- | --- | --- | --- | --- | --- | --- | --- |
| 1 | serous | 1 | 2 | IV | Pleura | 5FU, holoxan, cisplatin | CR |
| 2 | serous | 1 | 3 | Ib | no | Taxol, cisplatin | CR |
| 3 | serous | 2 | 0 | IIIb | Liver, lymph node | 5FU, holoxan, cisplatin | CR |
| 4 | serous | 2 | 0 | IIIc | Peritoneal carcinosis | 5FU, holoxan, cisplatin | PR |
| 5 | serous | 2 | 0 | IIIc | Peritoneal carcinosis | 5FU, holoxan, cisplatin | CR |
| 6 | serous | 2 | 1 | IIIc | Peritoneal carcinosis | 5FU, holoxan, cisplatin | CR |
| 7 | serous | 2 | 2 | IIIc | Peritoneal carcinosis | Taxol, cisplatin | CR |
| 8 | serous | 2 | 2 | IIIc | Pleura, liver | Carboplatin, taxol | CR |
| 9 | serous | 2 | 2 | IIb | no | 5FU, holoxan, cisplatin | - |
| 10 | serous | 2 | 3 | IV | Peritoneal carcinosis | MTX, endoxan, 5FU | CR |
| 11 | serous | 2 | 3 | Ib | no | 5FU, holoxan, cisplatin | CR |
| 12 | serous | 3 | 0 | IV | Pleura, lymph node | 5FU, holoxan, cisplatin | CR |
| 13 | serous | 3 | 0 | IIIc | no | 5FU, holoxan, cisplatin | PR |
| 14 | serous | 3 | 0 |  | Peritoneal carcinosis |  |  |
| 15 | serous | 3 | 0 | IIIc | Colon | 5FU, holoxan, cisplatin | PR |
| 16 | serous | 3 | 0 | IIIc | no | MTX, endoxan, 5FU | TP |
| 17 | serous | 3 | 0 | IIIc | Peritoneal carcinosis | 5FU, holoxan, cisplatin | CR |
| 18 | serous | 3 | 0 |  |  |  |  |
| 19 | serous | 3 | 0 | IIIc | Pelvis, lymph node | Taxol, cisplatin | PR |
| 20 | serous | 3 | 0 |  |  |  |  |
| 21 | serous | 3 | 1 | IV | Peritoneal c., pleura | 5FU, holoxan, cisplatin | CR |
| 22 | serous | 3 | 1 | IIIc | Colon | MTX, endoxan, 5FU | CR |
| 23 | serous | 3 | 1 |  |  |  |  |
| 24 | serous | 3 | 1 | IIIc | Peritoneal carcinosis | 5FU, holoxan, cisplatin | TP |
| 25 | serous | 3 | 1 | IV | Peritoneal carcinosis | Taxol, carboplatin | PR |
| 26 | serous | 3 | 1 | IIIc | Peritoneal carcinosis | Taxol, cisplatin | CR |
| 27 | serous | 3 | 1 | Ic | Peritoneal carcinosis | Taxol, cisplatin | CR |
| 28 | serous | 3 | 2 | IIIc | Lymph node | 5FU, holoxan, cisplatin | CR |
| 29 | serous | 3 | 2 | IIIc | Pelvis | 5FU, holoxan, cisplatin | CR |
| 30 | serous | 3 | 2 | IIIc | Brain | 5FU, holoxan, cisplatin | CR |
| 31 | serous | 3 | 2 | IIIc | Peritoneal carcinosis | Taxol, cisplatin | CR |
| 32 | serous | 3 | 2 | IIIc | ? (High CA125) | Taxol, cisplatin | PR |
| 33 | serous | 3 | 2 | IIIc | ? (High CA125) | Taxol, cisplatin | PR |
| 34 | serous | 3 | 2 | IIc | Pelvis | Taxol, cisplatin | CR |
| 35 | serous | 3 | 3 | IIIc | Peritoneal carcinosis | 5FU, holoxan, cisplatin | CR |
| 36 | serous | 3 | 3 | IIIc | Peritoneal carcinosis | Alkeran | TP |
| 37 | serous | 3 | 3 | IIIc | Liver, spleen | Taxol, cisplatin | CR |
| 38 | serous | 3 | 3 | IIb | Lymph node | 5FU, holoxan, cisplatin | CR |
| 39 | serous | 3 | 3 | IV | Peritoneal carcinosis | Therapeutic abstention | - |
| 40 | endometrioid | 2 | 0 |  |  |  |  |
| 41 | endometrioid | 2 | 0 | IIc | Peritoneal carcinosis | 5FU, holoxan, cisplatin | CR |
| 42 | endometrioid | 2 | 2 | IIb | no | 5FU, VP16, cisplatin | CR |
| 43 | endometrioid | 3 | 0 | IIIc | Peritoneal carcinosis | MTX, endoxan, velbe | SR |
| 44 | endometrioid | 3 | 1 | IIIc | no | Taxol, cisplatin | CR |
| 45 | mucinous | 1 | 0 | IIIc | Peritoneal carcinosis | 5FU, holoxan, cisplatin | TP |
| 46 | mucinous | 2 | 0 | Ic | Peritoneal carcinosis |  | CR |
| 47 | mucinous | 3 | 2 | IIIc | Peritoneal carcinosis | Taxol, cisplatin | CR |
| 48 | clear cells | 3 | 2 | Ic | no |  |  |
| 49 | other | 3 | 0 |  | adenocarcinome |  |  |
| 50 | other | 3 | 0 | IIIc | pelvis | 5FU, holoxan, cisplatin | TP |
