# Supplementary material for: New Blocking Antibodies Impede Adhesion, Migration and Survival of Ovarian Cancer Cells, Highlighting MFGE8 as a Potential Therapeutic Target of Human Ovarian Carcinoma
Source: PLoS One. 2013 Aug 16;8(8):e72708. doi: 10.1371/journal.pone.0072708 (PMC3745384; doi:10.1371/journal.pone.0072708)
Supplement: Table S2 — List of breast carcinoma biopsies used to generate the tumor microarray used for histology. (DOCX) [file pone.0072708.s002.docx]

**Table S2 : List of breast carcinoma biopsies used to generate the tumor microarray used for histology**

| **TMA sample** | **Phenotype** | **Grade** | **MFGE8 expression** |
| --- | --- | --- | --- |
| 1 | HR+/Her2+ | 1 | 0 |
| 2 | HR+/Her2+ | 2 | 0 |
| 3 | HR+/Her2+ | 2 | 0 |
| 4 | HR+/Her2+ | 2 | 1 |
| 5 | HR+/Her2- | 0 | 0 |
| 6 | HR+/Her2- | 0 | 1 |
| 7 | HR+/Her2- | 1 | 0 |
| 8 | HR+/Her2- | 1 | 0 |
| 9 | HR+/Her2- | 1 | 0 |
| 10 | HR+/Her2- | 2 | 0 |
| 11 | HR+/Her2- | 2 | 0 |
| 12 | HR+/Her2- | 2 | 0 |
| 13 | HR+/Her2- | 2 | 0 |
| 14 | HR+/Her2- | 2 | 0 |
| 15 | HR+/Her2- | 2 | 0 |
| 16 | HR+/Her2- | 2 | 0 |
| 17 | HR+/Her2- | 2 | 0 |
| 18 | HR+/Her2- | 2 | 1 |
| 19 | HR+/Her2- | 2 | 1 |
| 20 | HR+/Her2- | 2 | 1 |
| 21 | HR+/Her2- | 2 | 1 |
| 22 | HR+/Her2- | 2 | 1 |
| 23 | HR+/Her2- | 2 | 1 |
| 24 | HR+/Her2- | 2 | 1 |
| 25 | HR+/Her2- | 2 | 1 |
| 26 | HR+/Her2- | 2 | 1 |
| 27 | HR+/Her2- | 2 | 1 |
| 28 | HR+/Her2- | 2 | 2 |
| 29 | HR+/Her2- | 2 | 2 |
| 30 | HR+/Her2- | 2 | 3 |
| 31 | HR+/Her2- | 3 | 0 |
| 32 | HR+/Her2- | 3 | 0 |
| 33 | HR+/Her2- | 3 | 0 |
| 34 | HR+/Her2- | 3 | 1 |
| 35 | HR+/Her2- | 3 | 1 |
| 36 | HR+/Her2- | 3 | 1 |
| 37 | HR+/Her2- | 3 | 2 |
| 38 | HR-/Her2+ | 3 | 1 |
| 39 | HR-/Her2+ | 3 | 2 |
| 40 | HR-/Her2- | 3 | 0 |
| 41 | HR-/Her2- | 3 | 0 |
| 42 | HR-/Her2- | 3 | 0 |
| 43 | HR-/Her2- | 3 | 0 |
| 44 | HR-/Her2- | 3 | 0 |
| 45 | HR-/Her2- | 3 | 1 |
| 46 | HR-/Her2- | 3 | 1 |
| 47 | HR-/Her2- | 3 | 2 |
| 48 | HR-/Her2- | 3 | 2 |
| 49 | HR-/Her2- | 3 | 2 |
| 50 | HR-/Her2- | 3 | 2 |
| 51 | HR-/Her2- | 3 | 2 |
| 52 | HR-/Her2- | 3 | 2 |
| 53 | HR-/Her2- | 3 | 2 |
| 54 | HR-/Her2- | 3 | 2 |
| 55 | HR-/Her2- | 3 | 2 |
| 56 | HR-/Her2- | 3 | 2 |
| 57 | HR-/Her2- | 3 | 3 |
| 58 | HR-/Her2- | 3 | 3 |
| 59 | HR-/Her2- | 3 | 3 |
